# Supplementary material for: Exploring attitudes to decolonising the science curriculum—A UK Higher Education case study
Source: PLoS One. 2024 Nov 27;19(11):e0312586. doi: 10.1371/journal.pone.0312586 (PMC11602068; doi:10.1371/journal.pone.0312586)

**Supplementary Materials for:**

Towards Fairer Science Curricula – Exploring Attitudes, Misconceptions, and Barriers to Decolonising Higher Education

By: Lena Grinsted, Catherine Murgatroyd, and Jodi Burkett

**SM1**: The full questionnaire

Contents

[The full questionnaire 2](#_Toc121407182)

[Section 1: Consent 2](#_Toc121407183)

[Section 2: Participant demographics 2](#_Toc121407184)

[Section 3a: Familiarity with and understanding of decolonisation 3](#_Toc121407185)

[Section 3b: Benefits and risks 5](#_Toc121407186)

[Section 3c: Topics of training 6](#_Toc121407187)

[Section 4: Teaching activities 7](#_Toc121407188)

[Section 5: Importance, responsibility and barriers 10](#_Toc121407189)

# The full questionnaire

##

## Section 1: Consent


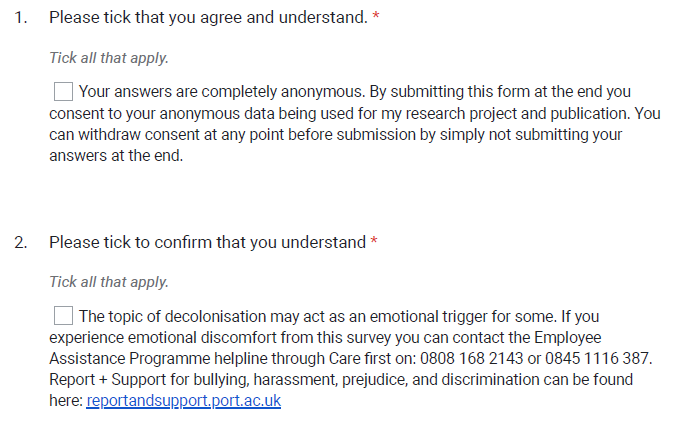


## Section 2: Participant demographics


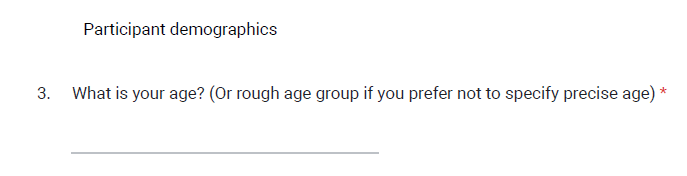


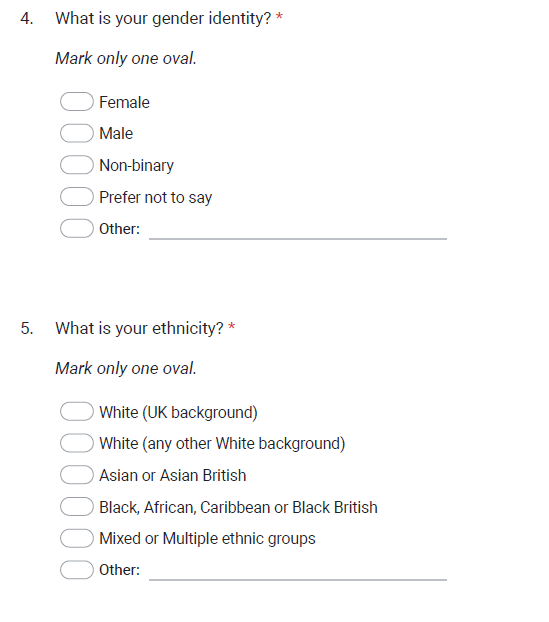


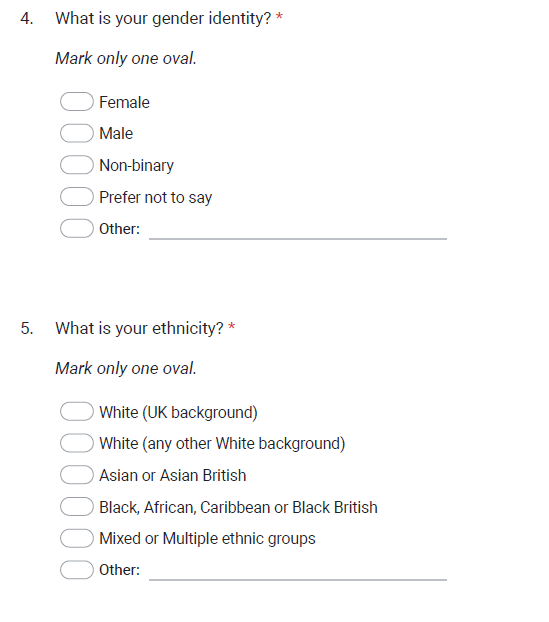


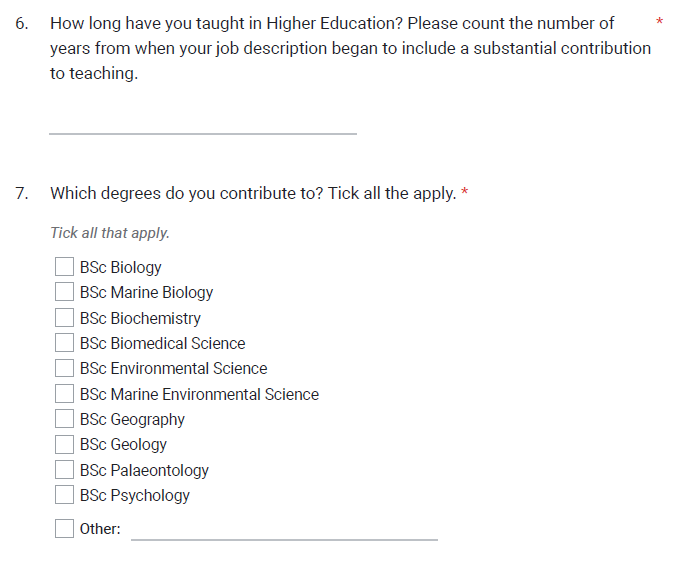


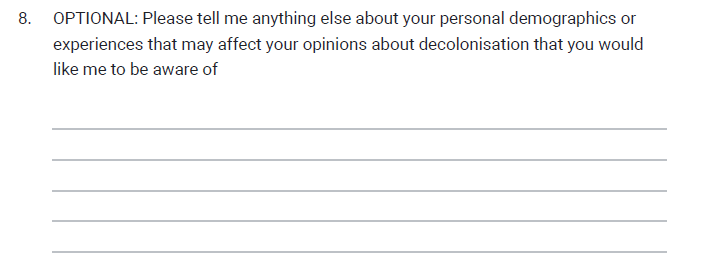


## Section 3a: Familiarity with and understanding of decolonisation


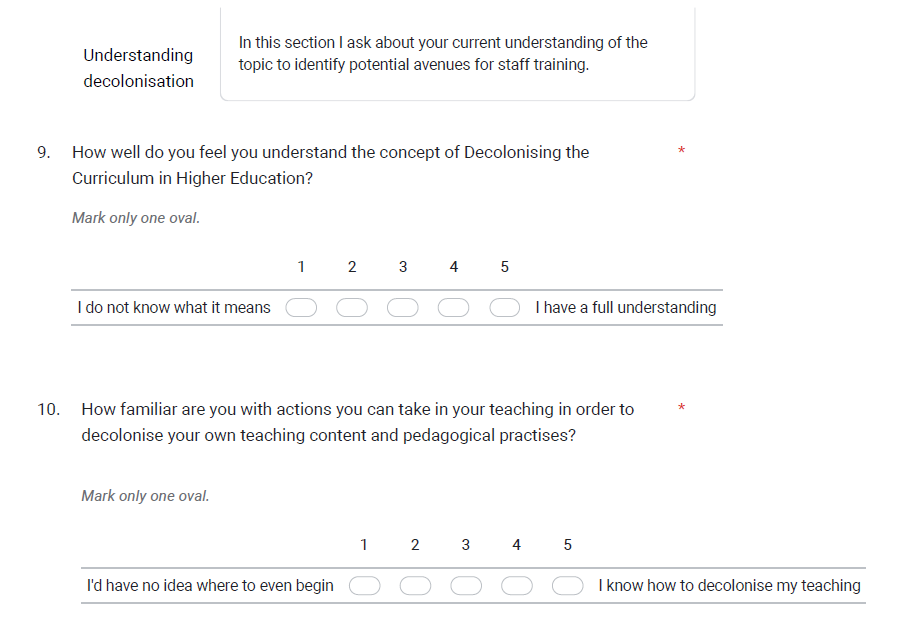


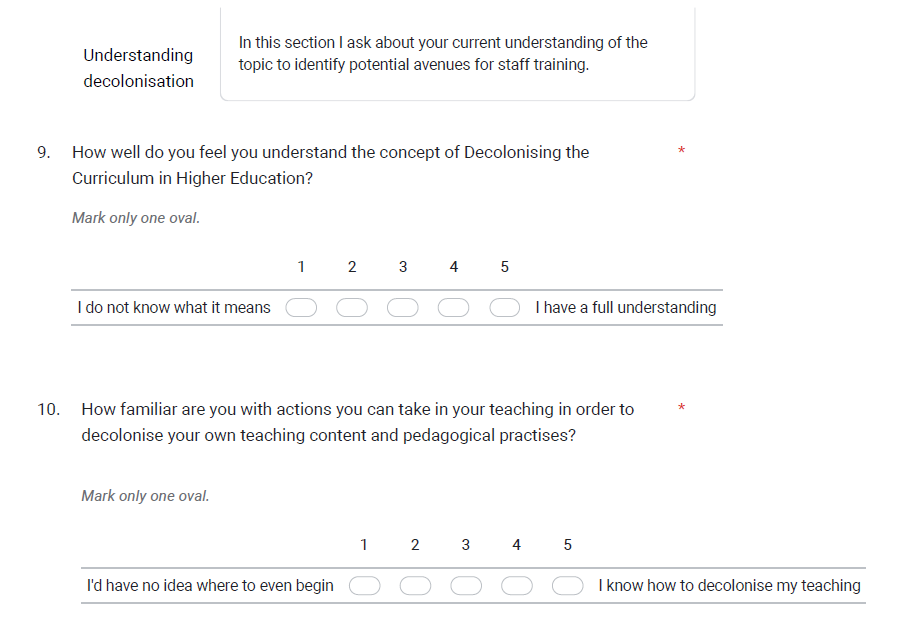


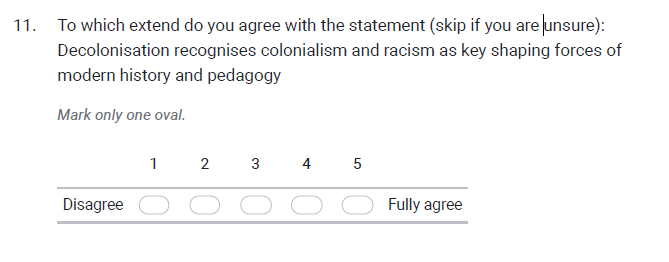


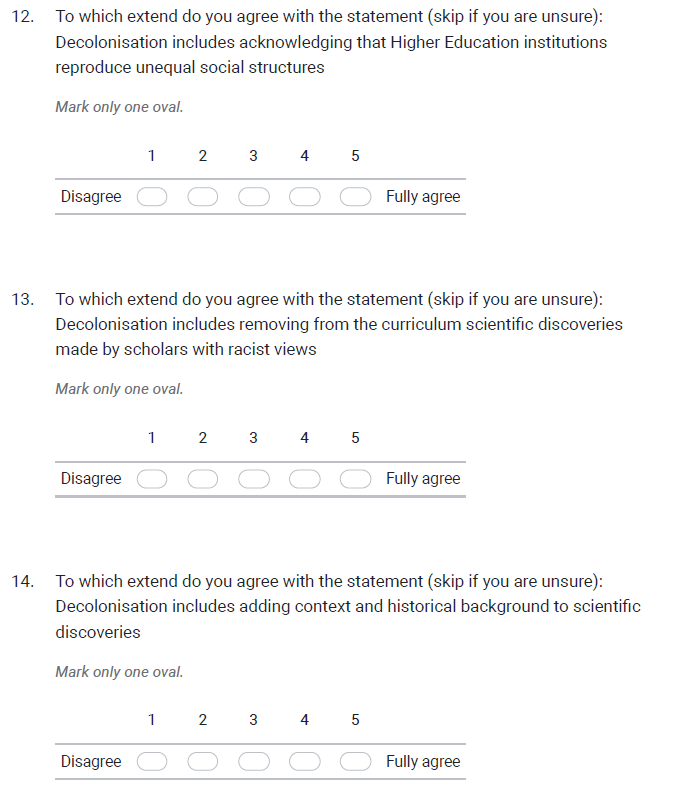


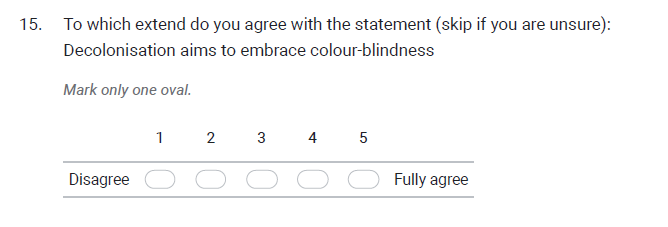


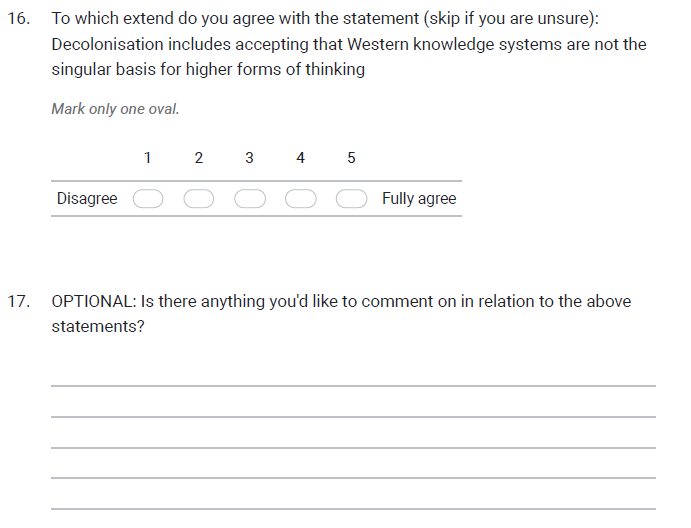


## Section 3b: Benefits and risks


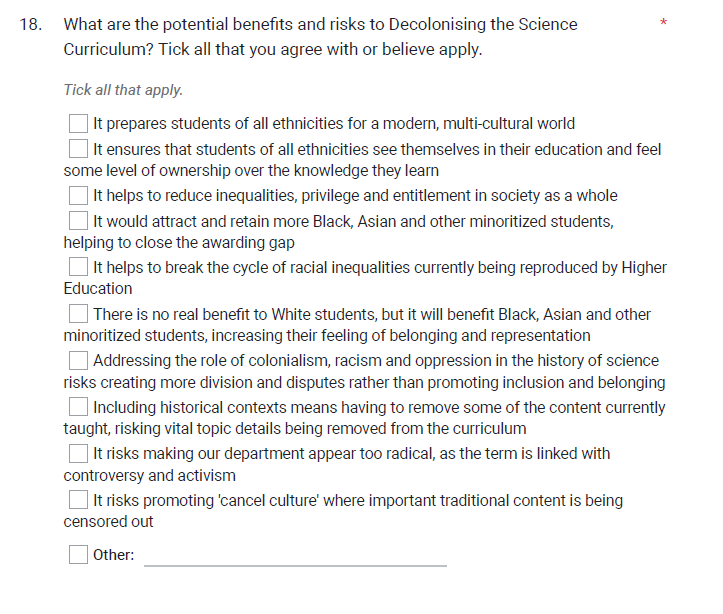


## Section 3c: Topics of training


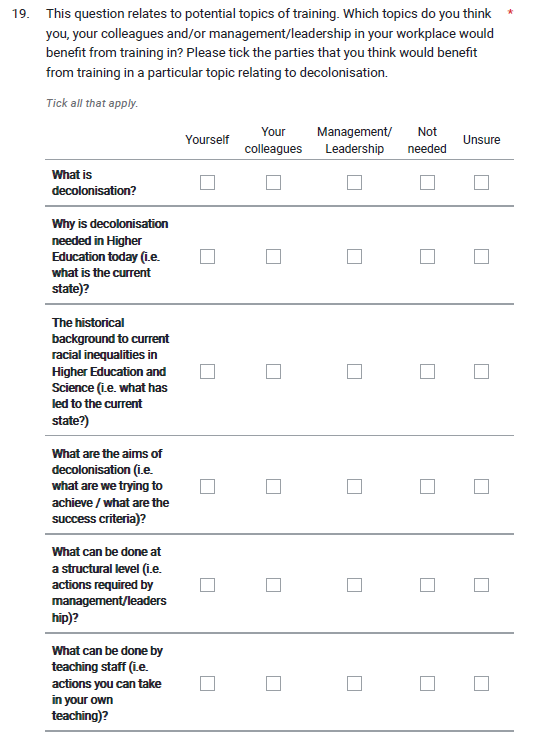


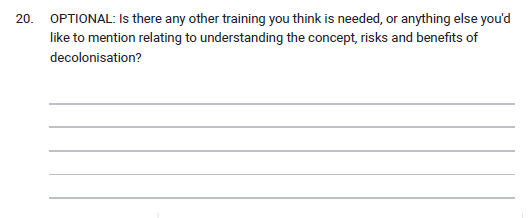


## Section 4: Teaching activities


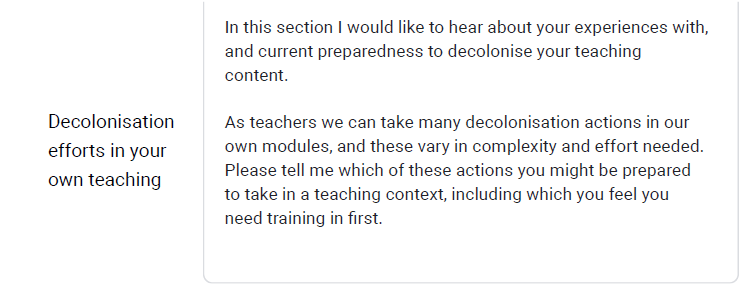


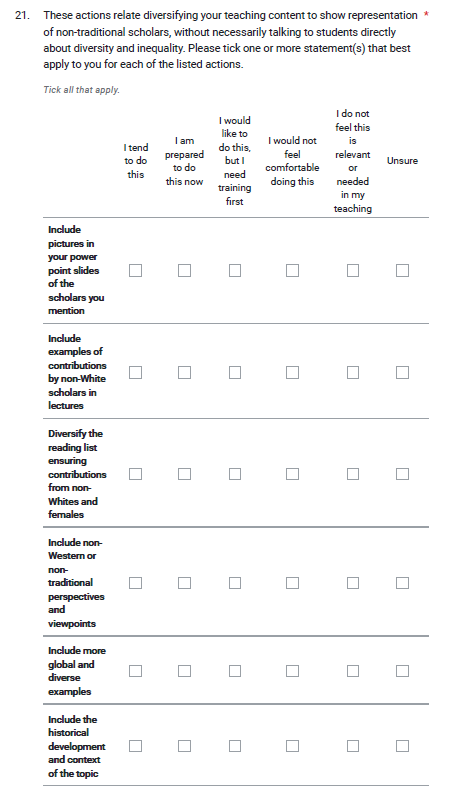


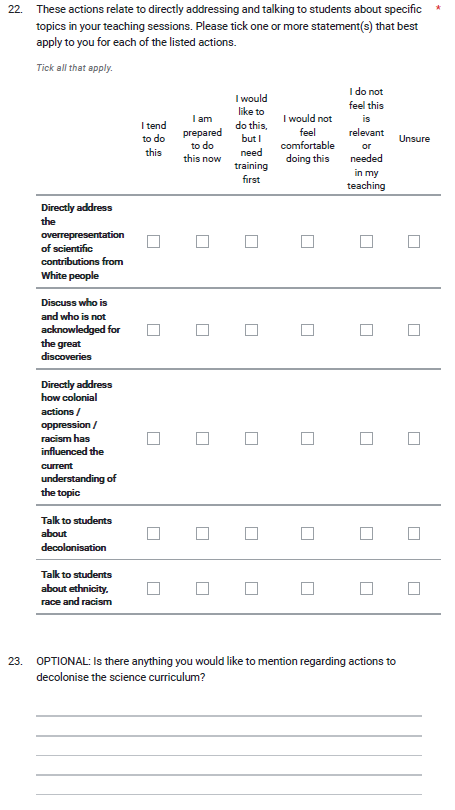


## Section 5: Importance, responsibility and barriers


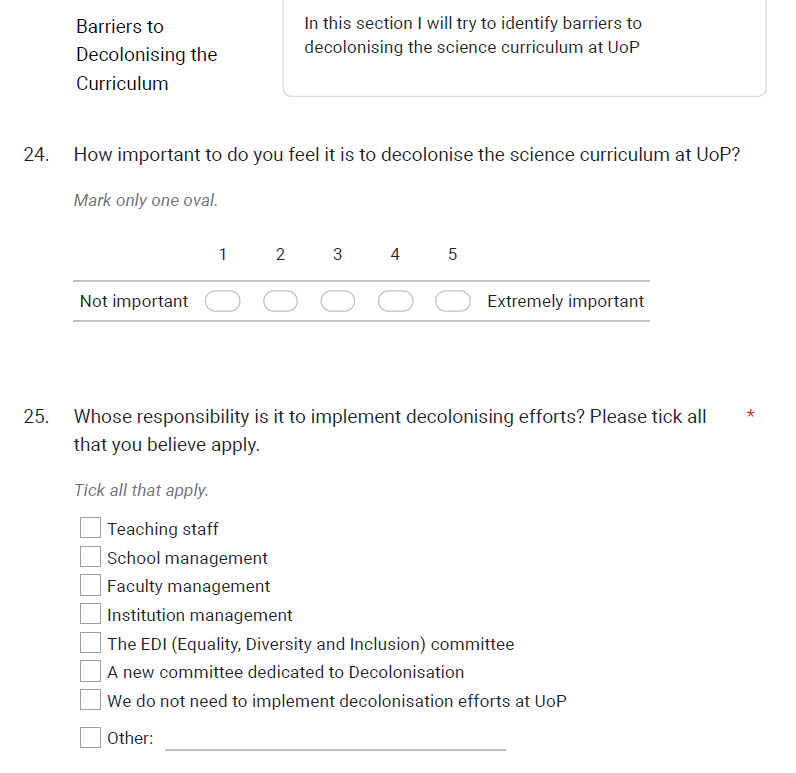


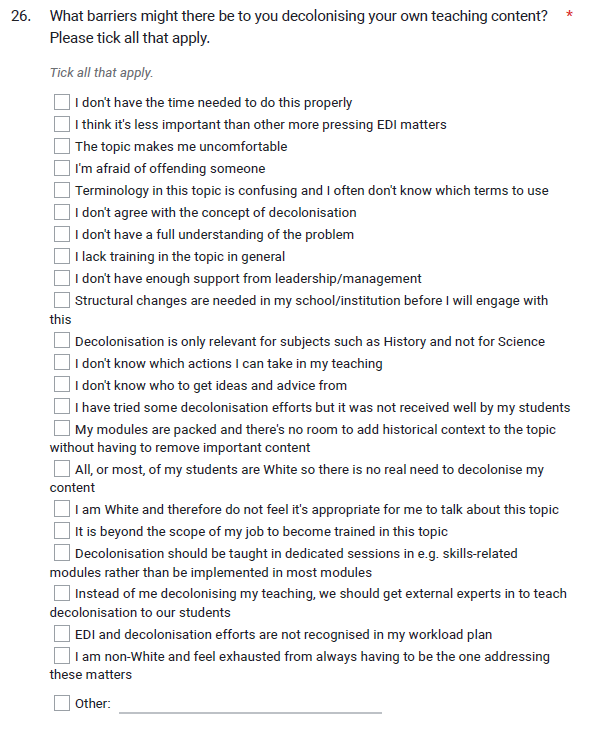


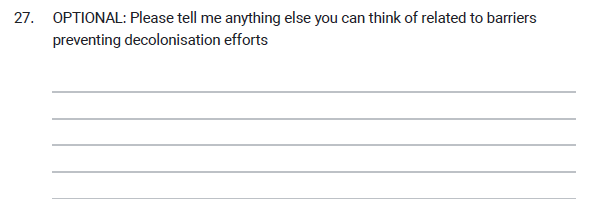

Supplement: S1 Appendix — (DOCX) [file pone.0312586.s001.docx]
