# Supplementary material for: Exploring attitudes to decolonising the science curriculum—A UK Higher Education case study
Source: PLoS One. 2024 Nov 27;19(11):e0312586. doi: 10.1371/journal.pone.0312586 (PMC11602068; doi:10.1371/journal.pone.0312586)
Supplement: S2 Appendix — Supplementary figures S1 Fig: Correlation between age and number of years in HES2 Fig: Degrees contributed toS3-S5 Figs: Effects of age, gender and discipline on perceptionsS6 Fig: Pairwise comparisons of types of teaching activitiesSupplementary tables S1 Table: Benefits and risksS2 Table: ResponsibilitiesS3 Table: Barriers (DOCX) [file pone.0312586.s002.docx]

**Supporting information for:**

Exploring attitudes to decolonising the science curriculum – a UK Higher Education case study

By: Lena Grinsted, Catherine Murgatroyd, and Jodi Burkett

**Appendix 2**: Supplementary figures and tables

Contents

[Supplementary figures 2](#_Toc157698173)

[Fig. S1: Correlation between age and number of years in HE 2](#_Toc157698174)

[Fig. S2: Degrees contributed to 2](#_Toc157698175)

[Figs S3-S5: Effects of age, gender and discipline on perceptions 3](#_Toc157698176)

[Fig. S6: Pairwise comparisons of types of teaching activities 4](#_Toc157698177)

[Supplementary tables 5](#_Toc157698178)

[Table S1: Benefits and risks 5](#_Toc157698179)

[Table S2: Responsibilities 5](#_Toc157698180)

[Table S3: Barriers 6](#_Toc157698181)

# Supplementary figures

##

## Fig. S1: Correlation between age and number of years in HE


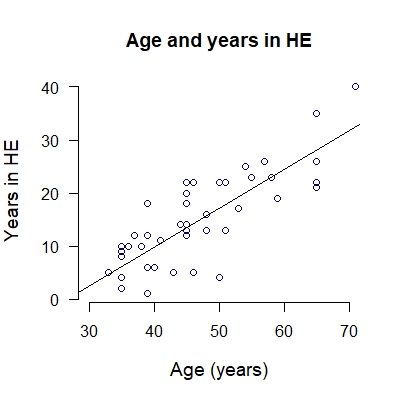


**Fig. S1** Correlation between age, in years, and number of years teaching in HE.

## Fig. S2: Degrees contributed to


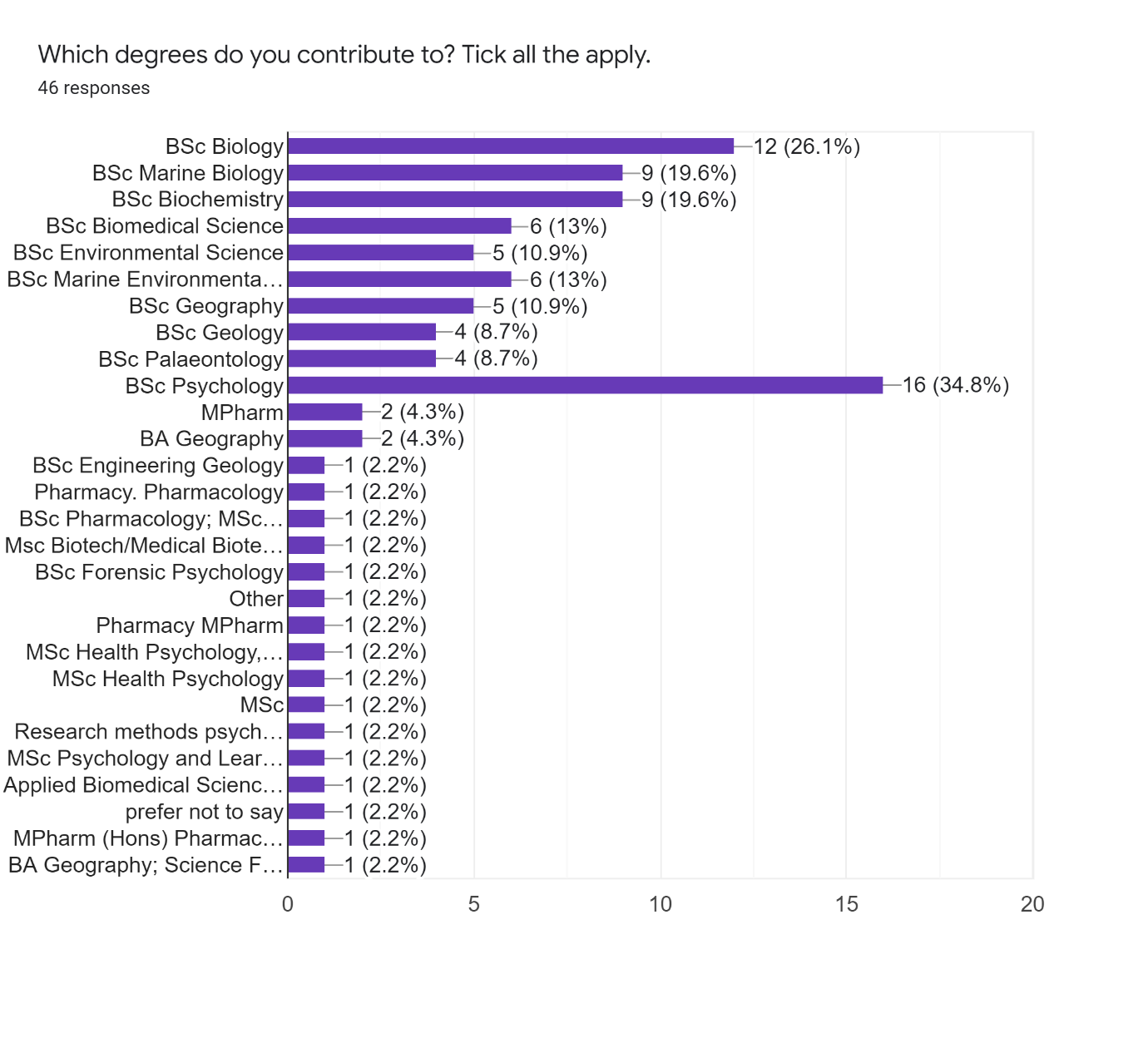


**Fig. S2** Number of respondents indicating which degrees they contributed to.

## Figs S3-S5: Effects of age, gender and discipline on perceptions


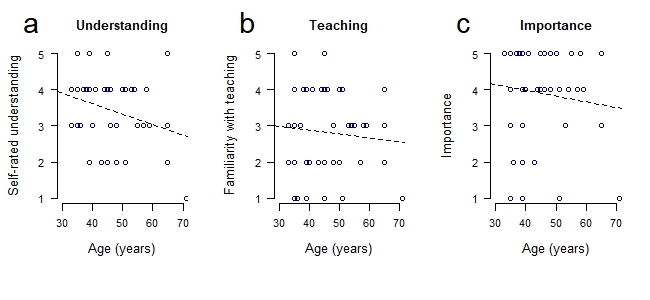


**Fig. S3** The effect of age on a) self-assessed understanding of Decolonisation, b) how familiar participants felt with teaching actions to Decolonise their content, and c) rating of how important participants felt it is to Decolonise the science curriculum. Stippled linear regression lines indicate non-significant non-parametric correlations.


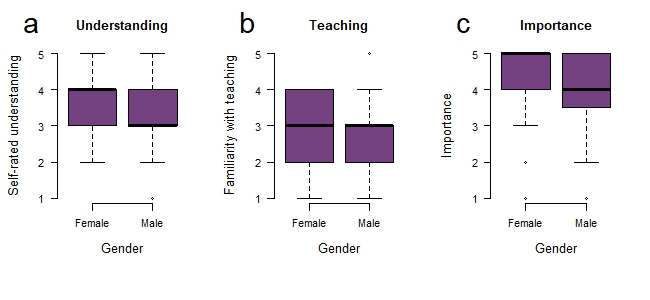
**Fig. S4** The effect of gender on a) self-assessed understanding of Decolonisation, b) how familiar participants felt with teaching actions to Decolonise their content, and c) rating of how important participants felt it is to Decolonise the science curriculum. Non-significance was found throughout.


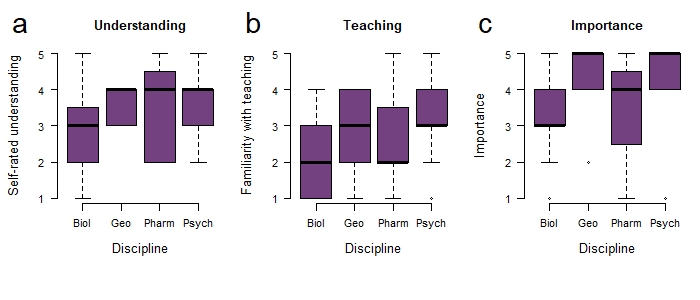
**Fig. S5** The effect of Discipline on a) self-assessed understanding of Decolonisation, b) how familiar participants felt with teaching actions to Decolonise their content, and c) rating of how important participants felt it is to Decolonise the science curriculum. Non-significance was found throughout.

## Fig. S6: Pairwise comparisons of types of teaching activities


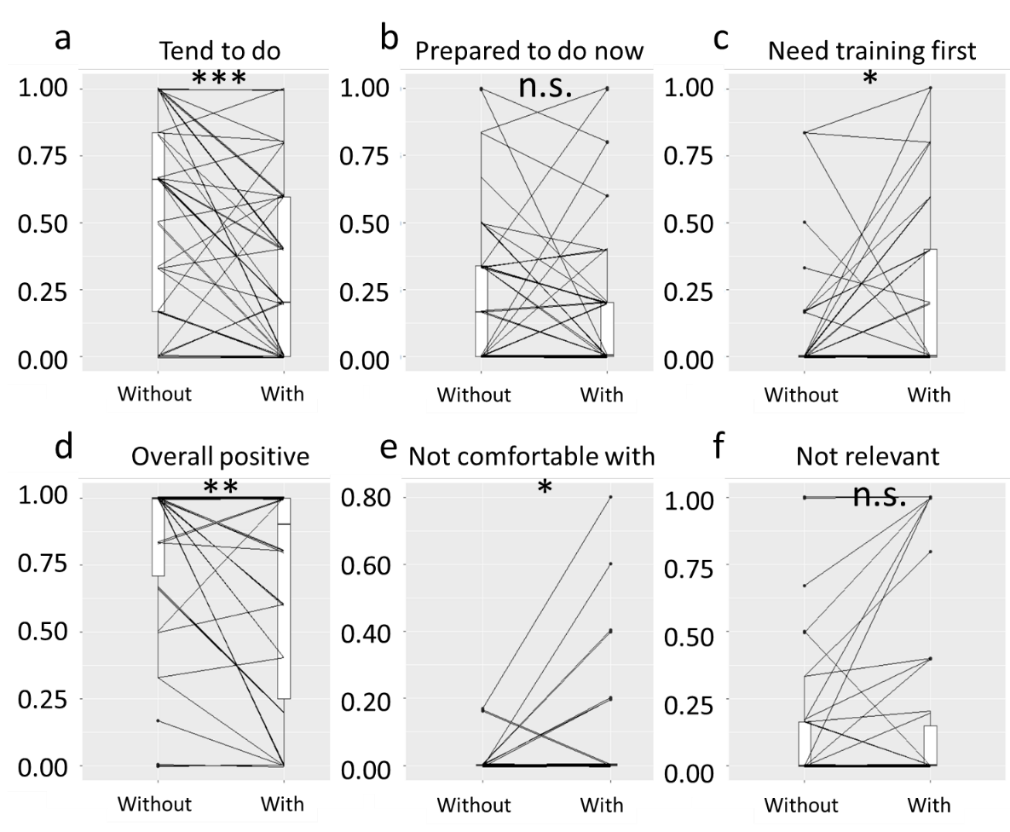


**Fig. S7** Box-and-whiskers pairwise comparisons between teaching activities *without* and *with* directly discussion topics related to Decolonisation in participants’ likelihood (from 0 to 1) of a) tending to do them, b) being prepared to do them now, c) wanting to do them but only after receiving training, d) being generally positive about the activities (responding either with (a), (b) or (c)), e) not being comfortable doing them, and f) not finding them relevant. Stars and n.s. indicate level of or absence of significance.

# Supplementary tables

##

## Table S1: Benefits and risks

**Table S1** Additional risks, benefits and comments added in the ‘Other’ option in the question about benefits and risks and benefits of Decolonisation.

| **Benefit or Risk** | **Additional benefits and risks suggested in the 'Other' option** |
| --- | --- |
| **Benefit** | It can improve knowledge and science as such, by showing how we can go wrong |
| **Benefit** | It will help open everyone's eyes to the past to help us learn and grow |
| **Benefit** | Decolonialisation will be beneficial for students, too, as all will see how scientific content and interpretation thereof is linked to ideology and politics. |
| **Benefit** | It acknowledges and reduces the impact of "white privilege" on the content in education |
| **Risk** | Decolonization theory is a pernicious ideology that will likely lead to universities losing the respect of parents, students, and employers. It is derived from an, critical theory unhealthy mix of Marxism, and postmodernism. I would consider moving to a university that does not promote such ideology. |
| **Risk** | Risks exist, but it should be possible to mitigate. |
| **NA** | this is just too simplistic |
| **NA** | all statements/questions assume the colonial past has some impact on education - not sure it does |
| **NA** | I don't really know what benefits and risks (if any) there are |

## Table S2: Responsibilities

**Table S2** Additional comments added in the ‘Other’ option in the question about whose responsibility it is to Decolonise the curriculum.

| **Additional responsibilities suggested in the 'Other' option** |
| --- |
| ALL |
| The curriculum is already decolonized. |
| All but teaching staff need support that currently uni does not offer with the contempt that they treat our time |
| This is nothing to be IMPLEMENTED - it is good to start a conversation, but nothing should be IMPLEMENTED (usually this is top-down anyway, which will make matters worse) |

## Table S3: Barriers

**Table S3** Additional comments added in the ‘Other’ option in the question about barriers to Decolonising the curriculum.

| **Additional barriers suggested in the 'Other' option** |
| --- |
| None of the above at all!!! |
| I am not certain what needs decolonizing. |
| I don't have a clear understanding of what decolonisation means (and to what extent I should regard it as a 'problem' that I have to resolve); at the moment it feels rather that the uni has discovered it as another box to tick, and that in and of itself doesn't convince me that it is a problem ... |
| It’s easy to say we will try to decolonise the curriculum, but most of the staff have been taught and learned the current curriculum so currently I do not feel I know where to start and more importantly I will need a lot of time to immerse myself in that literature to get a good understanding and not to allow my bias from my current knowledge affect my interpretations |
| WORKLOAD - it takes an enormous time to divert from a main textbook (which is definitely WEIRD & most White), search for new material, incorporate it in an equity manner (to be undermined by the simplicity of other lectures that only present the biased material. |
| I would need considerable time to research non-white resources in my subject areas. I would like to collaborate with historians and experts in humanities on that |
| It is often hard to actually find information relating to contributions of people from other cultures to my field. |
| I am a white male, and feel constantly under pressure and under scrutiny to be apologetic for the actions for my historical demographic (despite not being racist/misogynistic, and maing every effort to bring diversity into my teaching) |
| This is critical race praxis, and this ideology has to be challenged and kept out of rational empiricism in universities. |
