# Supplementary material for: Exploring attitudes to decolonising the science curriculum—A UK Higher Education case study
Source: PLoS One. 2024 Nov 27;19(11):e0312586. doi: 10.1371/journal.pone.0312586 (PMC11602068; doi:10.1371/journal.pone.0312586)
Supplement: S5 Appendix — (DOCX) [file pone.0312586.s005.docx]

**Abstract in Spanish and English**

Exploring attitudes to decolonising the science curriculum – a UK Higher Education case study

By: Lena Grinsted, Catherine Murgatroyd and Jodi Burkett

**Resumen científico Español:**

Los avances científicos están históricamente vinculados a las acciones coloniales de los imperios del pasado. El resultado es una producción de conocimiento sesgada hacia el mundo Occidental y con una representación mínima de académicos no-blancos en los planes del estudio de las Ciencias en la Educación Superior (ES). Los llamados a descolonizar los currículos de las ciencias buscan diversificar el contenido y reconocer el papel del racismo y el privilegio en la historia de la ciencia para crear una ES que aísle menos a las identidades minoritarias y sea mas acogedora para los estudiantes de todas las etnias. Este estudio investiga la familiaridad del personal docente con los conceptos erróneos sobre la descolonización en una institución de educación superior del Reino Unido mediante un cuestionario en línea. Además, evaluamos las percepciones de los participantes sobre las barreras, los beneficios y los riesgos, las necesidades de capacitación y la preparación para tomar medidas en su enseñanza. La mayoría de los participantes investigados tenían una disposición positiva hacia la descolonización de su curriculo. Tambien identificamos situaciones en las que conceptos erróneos vinculan la descolonización con el comportamiento de la llamada "cultura de cancelación " y "daltonismo cultural" eran comúnes, mientras que varias barreras importantes como la falta de formación y las limitaciones de tiempo frenan el progreso. Brindamos recomendaciones específicas para la capacitación del personal y las actividades docentes y una breve reseña histórica relevante para las ciencias biológicas. Apoyando a los docentes, que forman a las futuras generaciones de científicos, a descolonizar el currículo, podemos mejorar la equidad en la educación superior, y la sociedad.

**Abstract in English**

Scientific advances are historically linked to colonial actions of past empires resulting in knowledge production biased towards the West with minimal representation of scholars of other ethnicities than White in science curricula in Higher Education (HE). Calls to decolonise science curricula seek to diversify content by acknowledging the role of racism and privilege in the history of science, aiming at creating a HE that is less isolating for minoritised ethnicities and feels welcoming to students of all identities. This case study explored science teaching staff’s familiarity with and misconceptions of decolonisation at a UK HE institution using an online questionnaire. We further assessed participants' perceptions of barriers, benefits and risks, training needs, and preparedness to take actions in their teaching. We found that a majority of participants had a positive disposition towards decolonising their teaching, but that critical misconceptions, e.g. linking decolonisation to ‘cancel culture’ and ‘colour-blind’ behaviour were common, while important barriers, e.g. a lack of training and constraints on time, halt progress. We provide specific recommendations for staff training and a brief historical background relevant to life sciences. By supporting teachers, that train future generations of scientists, to decolonise the curriculum we can improve equity in HE, academia, and society.
